# Tolvaptan therapy of Chinese cirrhotic patients with ascites after insufficient diuretic routine medication responses: a phase III clinical trial

**DOI:** 10.1186/s12876-020-01536-0

**Published:** 2020-11-19

**Authors:** Jieting Tang, Yongfeng Wang, Tao Han, Qing Mao, Jun Cheng, Huiguo Ding, Jia Shang, Qin Zhang, Junqi Niu, Feng Ji, Chengwei Chen, Jidong Jia, Xiangjun Jiang, Nonghua Lv, Yueqiu Gao, Zhenghua Wang, Zhong Wei, Yingxuan Chen, Minde Zeng, Yimin Mao

**Affiliations:** 1 School of Medicine Division of Gastroenterology and Hepatology, Key Laboratory of Gastroenterology and Hepatology, Ministry of Health, Renji Hospital, School of Medicine, Shanghai Jiao Tong University, Shanghai Institute of Digestive Disease, No. 145, Middle Shandong Road, Shanghai, 200001 China; 2 Tianjin Third Hospital, Tianjin, China; 3 Southwest Hospital, Chongqing, China; 4 Beijing Ditan Hospital, Capital Medical University, Beijing, China; 5 Beijing Youan Hospital, Capital Medical University, Beijing, China; 6 Henan Provincial Peoples Hospital, Zhengzhou, China; 7 Shanghai Public Health Clinical Center, Shanghai, China; 8 The First Hospital of Jilin University, Changchun, China; 9 The First Affiliated Hospital, Zhejiang University, Hangzhou, China; 10 85 Hospital of Peoples Liberation Army, Shanghai, China; 11 Beijing Friendship Hospital, Capital Medical University, Beijing, China; 12 Qingdao Municipal Hospital, Qingdao, China; 13 The First Affiliated Hospital of Nanchang University, Nanchang, China; 14 Shanghai Shuguang Hospital, Shanghai, China

**Keywords:** Liver cirrhosis, Cirrhotic patients, Tolvaptan, Ascites

## Abstract

**Background:**

To determine the safety and efficacy of different doses of tolvaptan for treating Chinese cirrhotic patients with or without hyponatraemia who still had ascites after routine therapy with diuretics.

**Methods:**

In the present placebo-controlled, randomized, double-blinded, multicentre clinical trial, patients with cirrhotic ascites who failed to adequately respond to a combination of an aldosterone antagonist plus an orally administered loop diuretic were randomly placed at a 4:2:1 ratio into 3 groups [the 15 mg/day tolvaptan group (N = 301), 7.5 mg/day tolvaptan group (N = 153) and placebo group (N = 76)] for 7 days of treatment. The effects and safety were evaluated on days 4 and 7. A change in body weight from baseline on day 7 of treatment was the primary endpoint.

**Results:**

The administration of 7.5 or 15 mg/day tolvaptan significantly decreased body weight from baseline on day 7 of treatment compared to that with placebo treatment (*P* = 0.026; *P* = 0.001). For the secondary endpoints, changes in abdominal circumference from baseline and improvements in ascites were markedly different in the treatment groups and the placebo group on day 7 (*P*_7*.*5_ = 0.05, *P*_15*.*0_ = 0.002 and *P*_7*.*5_ = 0.037, *P*_15*.*0_ = 0.003), but there was no difference between the 7.5 mg/day and 15 mg/day dosage groups. The 24-h cumulative urine volume was higher in the 7.5 mg/day and 15 mg/day tolvaptan groups than the placebo group (*P* = 0.002, *P* < 0.001) and was greater in the 15 mg/day tolvaptan group than the 7.5 mg/day tolvaptan group (*P* = 0.004). Sodium serum concentrations were higher in patients with hyponatraemia after tolvaptan treatment, with no significant difference between the two dosage groups. The incidence of serious adverse drug reactions was not different between the groups (*P* = 0.543).

**Conclusions:**

Tolvaptan treatment at 7.5 mg per day might be a good therapeutic choice for Chinese cirrhotic patients with ascites who did not achieve satisfactory clinical responses to previous treatment regimens with combination therapy with an aldosterone antagonist and an orally administered loop diuretic.

**Trial registration:**

NCT01349348. Retrospectively registered May 2011.

## Background

A very common complication of liver cirrhosis is ascites, which usually leads to a poor prognosis for patients [1]. The underlying pathophysiological mechanisms involved in cirrhotic ascites are complex and remain to be fully elucidated. It is known, however, that the activation of the renin–angiotensin–aldosterone system, sympathetic nervous system, and arginine-vasopressin interactions are intimately involved in the formation of ascites. Therefore, drugs that suppress these neurohormones should be used to treat cirrhotic ascites patients. Aldosterone antagonists, such as spironolactone, administered alone or together with a loop diuretic, such as furosemide, are recommended as the first-line treatment [2].

Unfortunately, a proportion of patients do not adequately respond to this combination therapy [3], and this conventional combination is associated with side effects such as renal failure, electrolyte disturbance, gynaecomastia and muscle cramps [4]. Tolvaptan, a vasopressin V2 receptor antagonist, has emerged as a new treatment choice for patients with ascites. Under the brand names of SAMSCA, JINARC, and JYNARQUE, tolvaptan has been licensed in many countries as therapy for euvolemic and hypervolemic hyponatraemia [5–7]. In September 2013, Japan approved 7.5 mg/day tolvaptan for the treatment of patients with ascites who failed to adequately respond to conventional diuretics [8]. The Japan Liver Cirrhosis guidelines recommend the treatment of ascites with a dosage range from 3.75 to 7.5 mg/day [9].

Tolvaptan was approved in China in September 2011 to treat hypervolemic and euvolemic hyponatraemia caused by liver cirrhosis, heart failure or syndrome of inappropriate antidiuretic hormone in a dose range of 15–60 mg/day [10]. From March 2009 to February 2010, China carried out a phase II ascites trial that showed that tolvaptan was effective and safe for treating Chinese ascites patients, with no differences between a 15 mg/day or 30 mg/day therapy regimen [11]. At the time of the initiation of this study (October 2010), tolvaptan had not been approved in Japan for the treatment of ascites. However, a dose finding trial in Japan revealed that compared to 15 or 30 mg/day, 7.5 mg/day tolvaptan produced maximal changes in body weight and abdominal circumference measurements with good patient tolerance [12]. The aims of our clinical trial were to confirm the safety and effectiveness of 7 days of tolvaptan therapy for cirrhotic patients with ascites in China who had insufficiently responded to standard diuretic therapy and to determine the optimal tolvaptan dose.

## Methods

### Trial setting

This phase 3 trial was performed at 39 centres in China between October 5, 2010, and January 20, 2012, and followed the principles outlined in the Declaration of Helsinki. The study was approved by the ethics committee of Shanghai Renji Hospital or the ethics committees of individual participating institutions. Informed signed consent forms were obtained from all subjects who agreed to be enrolled in the trial. The registration number of the clinical trial was NCT01349348.

### Trial population

*Inclusion criteria* Hospitalized cirrhotic patients (18–75 years old) who were clinically or pathologically diagnosed and still presented with ascites after routine treatment, including a combination of oral loop diuretics and aldosterone antagonists with fixed doses for at least 4 days.

*Exclusion criteria* Patients suffering from hepatic encephalopathy (coma classification ≥ stage 2), cancerous ascites, or uncontrolled spontaneous bacterial peritonitis; patients likely to have gastrointestinal bleeding during the trial; or those receiving albumin or other blood preparations. In addition, patients with anuria (less than 100 mL of urine per day) and patients with dysuria caused by urinary tract stenosis, calculi and tumours were also excluded. Detailed information about the exclusion criteria is available in Additional file 1.

### Trial design

The clinical trial consisted of a ≤ 10-day screening period, observations for 3 days before treatment, a 7-day treatment period, and a ≤ 14-day follow-up period. The doses and methods of administration of conventional diuretics remained unchanged for 4 days prior to the initiation of tolvaptan therapy until 1 day after the treatment period. Patients restricted their salt intake but not their water intake. Patients whose body weight before breakfast was stable (< ± 1.0 kg) were randomly allocated in a 1:2:4 ratio to receive placebo, 7.5 mg tolvaptan, or 15 mg tolvaptan once daily for 7 consecutive days. A preliminary randomized drug code was designated for each dose, and the drug trial manager allocated each patient a specific therapy code that corresponded to the trial drug code.

The sample size was based on the results of previous studies and met the Chinese health authority’s requirements. To date, this is the largest tolvaptan randomized controlled trial (RCT) of liver cirrhosis with a total of 535 randomization patients, with 76, 154 and 305 patients in the various groups.

Day 1 was defined as the period from the first administration until the second administration of tolvaptan. Days 2–7 were similarly defined. The primary evaluation time was day 7, but day 4 evaluation was added to look for potentially unresponsive cases or worsened cases in the placebo group. If this situation occurred, contingency methods such as albumin infusion or paracentesis were applied.

### Efficacy assessment

Since there was a correlation between changes in body weight and ascites volume in patients with cirrhosis of the liver [13], a change in body weight is widely accepted as a useful marker for significant improvements in ascites and hepatic oedema. An alteration in patient body weight from baseline to the last dosage day (day 7) was the primary endpoint.

A change in abdominal circumference was the secondary endpoint. Abdominal circumference was measured with the patient in the supine position, legs straight and relaxed. If the patient had difficulties with a supine position, a prone position was also acceptable. The abdomen was totally relaxed. A tape with scale was placed under the patient’s back, perpendicular to the spine at the level of the umbilicus, touching but not compressing the skin and without twisting. When the patient had resumed regular normal breathing in a calm and relaxed manner, a measurement was taken at the end phase of exhalation. The date and measured abdominal circumference (accurate to 0.1 cm) were recorded in the original patient chart. If the abdominal circumference was reduced by ≥ 2 cm, the ascites condition was considered to have improved; other measurements were considered to be no change (increase or a reduction of < 2 cm) and deterioration (increase of > 2 cm). The percentage of improved cases among the total cases was defined as the improvement rate.

For patients who had lower limb oedema at baseline, lower limb oedema improvement rates were also evaluated. We assessed the degree of lower limb oedema as none, mild, moderate or severe. Changes were characterized as markedly improved (completely resolved or improved by ≥ 2 grades), improved (≥ 1 grade), unchanged or worsened (by ≥ 1 grade). The percentage of greatly improved or improved cases at baseline was defined as the improvement rate.

Serum electrolyte concentrations (Na^+^ and K^+^) were measured at baseline and 4–8 h after the first test drug dose as well as on days 1, 4 and 7. Cumulative 24-h urine volume and fluid intake were recorded daily.

Most parameters were measured in the morning after urination but before breakfast.

### Safety assessments

Lab test results of renal and liver function were analysed at baseline and on days 4 and 7. Vital signs were recorded every day. A 12-lead electrocardiogram was recorded for each patient at baseline and on day 7. Adverse events were assessed during the whole study and followed up until they were resolved.

### Statistical analysis

ANOVA (linear model) was used to look for any differences in efficacy between the 2 tolvaptan dosage groups and the placebo group, and the respective 95% confidence intervals (CIs) were calculated. Moreover, the correlation coefficient between changes in abdominal circumference and body weight on day 7 was calculated for both tolvaptan dosage groups. The regression equation and correlation coefficient were evaluated. Continuous variables were evaluated using ANOVA, while categorical variables were compared using either the Fischer exact or Kruskal–Wallis rank-sum tests. A difference was considered statistically significant at a *P* value < 0.05 (two-sided). Statistical analyses were conducted using SAS (ver. 9.2; Cary, NC, US).

For efficacy analysis, all patients who were randomized in the trial were included in the full analysis set (FAS). Any missing value data in the FAS were replaced using the last observation carried forward (LOCF) algorithm. After each patient had completed or discontinued their participation in the trial, the time point was the end of treatment (EOT) 7 (+ 3) days after the last treatment.

## Results

### Clinical characteristics and demographic parameters of enrolled patients

Of the 639 enrolled patients from 39 centres who insufficiently responded to primary at least 4 days of combination therapy with routine diuretic treatments, 535 were eligible to participate in the trial, with 76, 154 and 305 patients randomly allocated to the placebo, 7.5 mg or 15 mg tolvaptan groups, respectively. One patient in each of the tolvaptan dosage groups did not receive the study drug, and 3 in the 15 mg tolvaptan dosage group were lost to follow-up (Additional file 1: Fig. 1). The baseline clinical and demographic characteristics of the trial patients are presented in Table 1. There were significant differences in body weight and abdominal circumference (*P* = 0.008 and *P* < 0.001), and most patients had hepatitis B (64.5–66.4%), were in the Child–Pugh class B or C (97.7–98.7%) and nearly 100% had ascites without hepatic encephalopathy. Biochemical measurements of serum creatinine (Cr), blood urea nitrogen (BUN) and albumin, as well as all other characteristics, were well balanced among the 3 groups of patients (Table 1).

**Table 1:** Clinical characteristics and demographic data at baseline (FAS)

Variables	Placebo (N = 76)	Tolvaptan 7.5 mg (N = 153)	Tolvaptan 15 mg (N = 301)	*P* value
Age (years, mean ± SD)	54.4 ± 12.3	53.8 ± 10.4	54.2 ± 10.9	0.847^†^
Gender male (N, %)	54 (71.1)	109 (71.2)	215 (71.4)	1.000^‡^
Body weight kg (mean ± SD)	63.5 ± 12.8	60.6 ± 10.1	62.9 ± 12.0	0.008^†^
Abdominal circumference (cm, mean ± SD)	87.8 ± 12.0	84.7 ± 9.0	87.9 ± 11.1	< 0.001^†^
Severity of lower limb edema (N, %)				0.837^§^
Non	50 (65.8)	106 (69.3)	212 (70.4)	
Mild	17 (22.4)	28 (18.3)	59 (19.6)	
Moderate	7 (9.2)	16 (10.5)	23 (7.6)	
Severe	2 (2.6)	3 (2.0)	7 (2.3)	
Duration of cirrhosis (day, mean ± SD)	842.8 ± 1137.5	916.8 ± 1622.6	894.8 ± 1460.9	0.925^†^
Etiology of liver cirrhosis (N, %)				0.371^§^
Hepatitis B	49 (64.5)	101 (66.0)	200 (66.4)	
Hepatitis C	9 (11.8)	9 (5.9)	18 (6.0)	
Alcoholic hepatitis	12 (15.8)	31 (20.3)	57 (18.9)	
Primary biliary cirrhosis	3 (3.9)	3 (2.0)	7 (2.3)	
Unknown	3 (3.9)	7 (4.6)	16 (5.3)	
Others	5 (6.6)	13 (8.5)	25 (8.3)	
Child–Pugh class (N, %)				0.702^§^
Class A	1 (1.3)	2 (1.3)	7 (2.3)	
Class B	48 (63.2)	96 (62.7)	190 (63.1)	
Class C	27 (35.5)	55 (35.9)	104 (34.6)	
Albumin concentration (g/dL, mean ± SD)	3.0 ± 0.4	3.1 ± 0.5	3.0 ± 0.5	0.218^†^
Albumin level (N, %)				0.430^§^
> 3.5 g/dL	8 (10.5)	30 (19.6)	52 (17.3)	
2.8–3.5 g/dL	48 (63.2)	85 (55.6)	161 (53.5)	
< 2.8 g/dL	20 (26.3)	38 (24.8)	88 (29.2)	
Serum sodium (mmol/L, mean ± SD)	137.7 ± 4.4	136.7 ± 5.1	136.9 ± 4.8	0.322^†^
Serum sodium < 135 mmol/L (N, %)	17 (22.4)	41 (26.8)	85 (28.2)	0.587^§^
Serum potassium (mmol/L, mean ± SD)	3.9 ± 0.5	4.1 ± 0.6	4.0 ± 0.5	0.022^†^
Scr (mg/dL, mean ± SD)	0.8 ± 0.2	0.8 ± 0.3	0.9 ± 0.3	0.276^†^
BUN (mmol/L, mean ± SD)	6.2 ± 3.0	6.3 ± 3.4	6.8 ± 3.6	0.264^†^
TB (µmol/L, mean ± SD)	43.8 ± 42.1	49.2 ± 57.2	43.6 ± 47.7	0.509^†^
AST (IU/L, mean ± SD)	67.4 ± 54.9	68.8 ± 56.9	66.0 ± 54.2	0.875^†^
ALT/GPT (IU/L, mean ± SD)	42.2 ± 27.1	43.1 ± 37.7	44.2 ± 39.3	0.899^†^
Dose of conventional diuretics				
Loop diuretics, furosemide equivalent (N, %)	73 (96.1)	144 (94.1)	278 (92.4)	0.583^§^
20–39 mg/day	22 (30.1)	44 (30.6)	78 (28.1)	
40–59 mg/day	23 (31.5)	51 (35.4)	106 (38.1)	
60–79 mg/day	11 (15.1)	18 (12.5)	36 (12.9)	
80–99 mg/day	8 (11.0)	17 (11.8)	37 (13.3)	
100 mg/day	9 (12.3)	14 (9.7)	21 (7.6)	
Aldosterone antagonist—spironolactone equivalent (N, %)	76 (100.0)	153 (100.0)	300 (99.7)	0.127^§^
20–39 mg/day	2 (2.6)	2 (1.3)	0 (0.0)	
40–59 mg/day	17 (22.4)	28 (18.3)	56 (18.7)	
60–79 mg/day	6 (7.9)	15 (9.8)	22 (7.3)	
80–99 mg/day	11 (14.5)	28 (18.3)	54 (18.0)	
100 mg/day	40 (52.6)	80 (52.3)	168 (56.0)	

Data are expressed as the mean ± SD or the number of patients (%)

Statistical analyses were conducted using ^†^ANOVA, ^‡^Fischer’s exact or ^§^Kruskal–Wallis rank sum tests

*ALT* alanine transaminase, *AST* aspartate transaminase, *BUN* blood urea nitrogen, *GPT* glutamic-pyruvic transaminase, *Scr* serum creatinine, *TB* total bilirubin

### Changes in body weight

The changes in body weight from baseline to day 7 were − 1.2 ± 2.2 kg in the placebo group, − 2.0 ± 2.4 kg in the 7.5 mg tolvaptan group and − 2.2 ± 2.5 kg in the 15 mg tolvaptan group (Table 2). The difference in body weight changes on day 7 for the 7.5 mg tolvaptan and placebo groups was − 0.8 kg (95% CI − 1.4 to − 0.1; *P* = 0.026) and − 1.0 kg for the 15 mg tolvaptan and the placebo groups (95% CI − 1.6 to − 0.4; *P* = 0.001) (Table 2). It is worth noting that there were no significant differences in body weight changes between the 7.5 mg and 15 mg tolvaptan groups at this time point.

**Table 2:** Change in body weight on day 7 as primary endpoint of the trial

	(N = 76)	Tolvaptan					*P* value		
		7.5 mg/day (N = 153)		15 mg/day (N = 301)		Difference between 7.5 mg/day and 15.0 mg/day, 95% CI			
	Mean ± SD	Mean ± SD	Difference from placebo, 95% CI	Mean ± SD	Difference from placebo, 95% CI		7.5 mg Tolvaptan versus placebo	15.0 mg Tolvaptan versus placebo	7.5 mg versus 15.0 mg Tolvaptan
Baseline	63.5 ± 12.8	60.6 ± 10.1		62.9 ± 12.0					
Day 7	62.3 ± 12.4	58.6 ± 10.1		60.8 ± 12.0					
Day 7—baseline	− 1.2 ± 2.2	− 2.0 ± 2.4	− 0.8 (− 1.4, − 0.1)	− 2.2 ± 2.5	− 1.0 (− 1.6, − 0.4)	− 0.2 (− 0.7, 0.3)	0.026	0.001	0.339

**Fig. 1:**
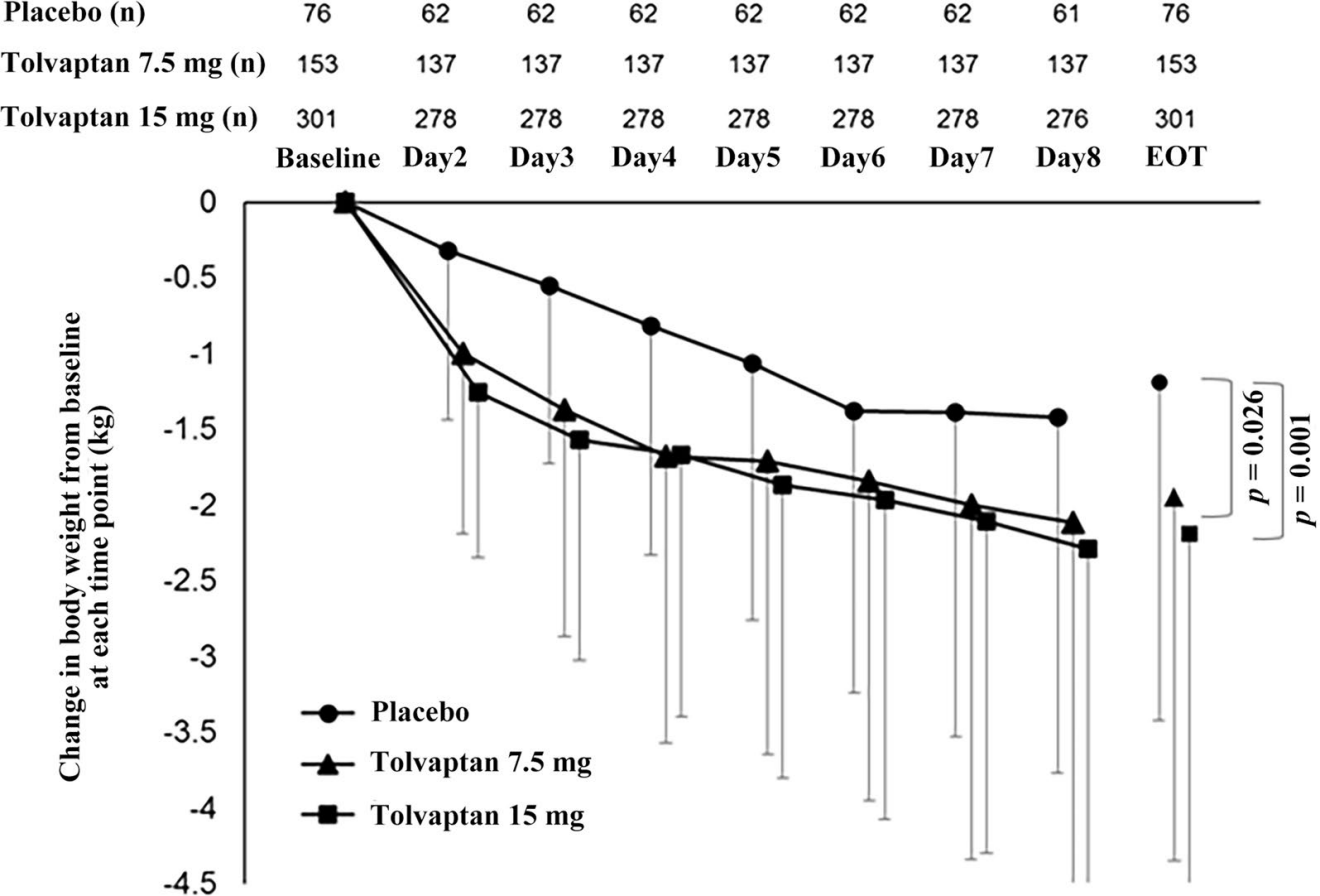
Change in body weight from baseline at each time point in the placebo, 7.5 mg and 15 mg tolvaptan groups. Data are expressed as the mean ± standard deviation (SD). The comparison between the tolvaptan and placebo groups was performed using ANOVA. End of treatment (EOT)

Daily changes in body weight showed significant differences for most time points between the placebo and the 7.5 mg and 15 mg tolvaptan groups (both *P* < 0.05) (Fig. 1, Additional file 1: Table 1).

### Analysis of factors predicting responsiveness

Patients who had a body weight loss > 1.5 kg during treatment were defined as responders. Linear regression analysis showed that tolvaptan treatment (*P* = 0.036 for 7.5 mg, *P* = 0.004 for 15 mg), baseline body weight (*P* = 0.019), baseline albumin < 2.8 g/dL (vs > 3.5 g/dL) (*P* = 0.016) and baseline BUN levels (*P* = 0.020) were associated with responsiveness (Additional file 1: Table 2). Multivariable regression analysis also showed that tolvaptan treatment (*P* = 0.017 for 7.5 mg vs placebo, *P* = 0.003 for 15 mg vs placebo), baseline body weight (*P* = 0.047), albumin levels < 2.8 g/dL (vs > 3.5 g/dL) (*P* = 0.027) and baseline BUN levels (*P* = 0.027) were significantly correlated with responsiveness (Table 3).

**Table 3:** Treatment effect predictors of body weight loss > 1.5 kg analyzed by a multivariable regression method

Variable	OR	95% CI	*P* value
Treatment group			
Tolvaptan 7.5 mg/day (vs Placebo)	2.028	1.1–3.6	0.017
Tolvaptan 15 mg/day (vs placebo)	2.280	1.3–3.9	0.003
Body weight (baseline, kg)	1.016	1.0–1.0	0.047
Albumin level (g/dL)			
2.8–3.5 (vs > 3.5)	1.395	0.8–2.3	0.949
< 2.8 (vs > 3.5)	1.900	1.1–3.3	0.027
BUN (baseline, mmol/L)	0.945	0.9–1.0	0.027

*BUN* blood urea nitrogen

### Improvement of ascites and lower extremity oedema

The change in the abdominal circumference from baseline to day 7 was − 1.7 ± 3.5 cm, − 2.7 ± 3.4 cm and − 3.2 ± 3.8 cm for the placebo, tolvaptan 7.5 mg and tolvaptan 15 mg groups, respectively. Compared to the placebo group, the 7.5 mg and 15 mg tolvaptan groups had significantly decreased abdominal circumference from baseline to day 4 and day 7 (*P* = 0.012 and *P* = 0.05, and *P* < 0.001 and *P* = 0.002). However, no significant difference was observed between the two tolvaptan groups.

Compared to the placebo group, the improvement rate of ascites in the 15 mg tolvaptan group was significantly higher on both day 4 and day 7 (*P* = 0.023 and 0.003) but was significant on only day 7 for the 7.5 mg tolvaptan group. This may imply that initiating treatment with a lower dose requires a longer time for improvements to be observed. The improvement rate of lower limb oedema was higher in the tolvaptan groups but was not significantly different compared to that in the placebo group. This was mainly because most patients did not have significant lower limb oedema (Table 4).

**Table 4:** The improvement of ascites and lower extremity edema (LOCF)

	Placebo (mg) (N = 76)				Tolvaptan						*P* value		
					7.5 mg/day (N = 153)			15 mg/day (N = 301)					
	Improvement		No changes	Degradation	Improvement	No changes	Degradation	Improvement	No changes	Degradation	7.5 mg Tolvaptan versus placebo	15.0 mg Tolvaptan versus placebo	7.5 mg versus 15.0 mg Tolvaptan
Improvement rate of ascites (N, %)													
Day 4		28 (36.8)	38 (50.0)	10 (13.2)	70 (45.8)	71 (46.4)	12 (7.8)	152 (50.5)	125 (41.5)	24 (8.0)	0.130	0.023	0.403
Day 7		31 (40.8)	34 (44.7)	11 (14.5)	82 (53.6)	60 (39.2)	11 (7.2)	180 (59.8)	93 (30.9)	28 (9.3)	0.037	0.003	0.342
Improvement rate of lower extremity edema (N, %)													
Day 4		12 (46.2)	12 (46.2)	2 (7.7)	23 (51.1)	21 (46.7)	1 (2.2)	48 (55.2)	39 (44.8)	0 (0.0)	0.395	0.223	0.766
Day 7		14 (53.8)	10 (38.5)	2 (7.7)	33 (71.7)	12 (26.1)	1 (2.2)	60 (67.4)	27 (30.3)	2 (2.2)	0.077	0.088	0.773

### Serum sodium and potassium concentrations

The serum Na^+^ concentration decreased from baseline in the placebo group, while in both tolvaptan dosage groups, the Na^+^ concentration significantly increased at each time point analysed (*P* < 0.001). The Na^+^ concentration increased more in the 15 mg tolvaptan group than in the 7.5 mg tolvaptan group, with significant differences on day 1 and day 4 (*P* < 0.001 and *P* = 0.002), but the difference gradually diminished by day 7 (*P* = 0.075) (Table 5).

**Table 5:** Change of serum sodium and potassium (all patients and hyponatremia patients)

		Placebo (mg)		Tolvaptan				*P* value		
				7.5 mg/day		15 mg/day				
		Mean ± SD	Changes from baseline	Mean ± SD	Changes from baseline	Mean ± SD	Changes from baseline	7.5 mg tolvaptan versus placebo	15.0 mg tolvaptan versus placebo	7.5 mg versus 15.0 mg tolvaptan
All patients (N)		76		153		301				
Serum Na^+^ concentration (mmol/L)	Baseline	137.7 ± 4.4	–	136.7 ± 5.1	–	136.9 ± 4.8	–	–	–	–
	4–8 h	136.2 ± 5.2	− 1.4 ± 3.1	137.7 ± 5.9	1.0 ± 3.6	138.2 ± 5.8	1.3 ± 3.8	< 0.001	< 0.001	0.408
	Day 1	137.6 ± 4.6	− 0.1 ± 2.8	138.8 ± 5.4	2.0 ± 3.4	140.3 ± 4.9	3.4 ± 3.4	< 0.001	< 0.001	< 0.001
	Day 4	137.4 ± 5.1	− 0.3 ± 3.14	138.3 ± 5.1	1.6 ± 3.0	139.6 ± 4.7	2.7 ± 3.9	< 0.001	< 0.001	0.002
	Day 7	137.0 ± 5.1	− 0.7 ± 3.4	138.2 ± 5.1	1.4 ± 3.3	139.0 ± 4.6	2.1 ± 4.0	< 0.001	< 0.001	0.075
Serum K^+^ concentration (mmol/L)	Baseline	3.9 ± 0.5	–	4.1 ± 0.6	–	4.0 ± 0.5	–	–	–	–
	Day 4	4.0 ± 0.7	0.1 ± 0.5	4.2 ± 0.4	0.1 ± 0.5	4.2 ± 0.7	0.1 ± 0.7	0.752	0.845	0.494
	Day 7	4.0 ± 0.5	0.1 ± 0.5	4.2 ± 0.5	0.1 ± 0.5	4.2 ± 0.5	0.2 ± 0.6	0.885	0.390	0.199
Hyponatremia patients (Na^+^ baseline< 135 mmol/L) (N)		17		41		85				
Serum Na^+^ concentration (mmol/L)	Baseline	131.3 ± 3.1	–	130.2 ± 4.5	–	130.9 ± 3.7	–	–	–	–
	4–8 h	129.4 ± 4.6	− 1.9 ± 3.7	131.1 ± 5.7	1.0 ± 3.9	132.7 ± 6.2	1.8 ± 4.3	0.019	< 0.001	0.260
	Day 1	132.6 ± 4.9	1.2 ± 3.7	133.5 ± 6.0	3.3 ± 3.8	135.6 ± 5.1	4.7 ± 3.7	0.061	< 0.001	0.057
	Day 4	131.2 ± 5.0	− 0.1 ± 4.6	133.2 ± 5.9	3.0 ± 3.2	135.4 ± 5.1	4.5 ± 4.6	0.013	< 0.001	0.063
	Day 7	130.3 ± 4.7	− 1.0 ± 4.1	132.8 ± 5.2	2.6 ± 3.2	134.9 ± 4.9	4.0 ± 5.1	0.005	< 0.001	0.144

This trend was also observed in hyponatraemia patients. Although 7.5 mg tolvaptan also increased sodium, overall sodium levels were still below 135 mmol/L at the end of treatment, while 15 mg tolvaptan normalized overall sodium levels starting on day 1. Comparing tolvaptan effects on hyponatraemia and normonatraemia patients, tolvaptan had stronger effects on hyponatraemia patients by increasing the absolute value of sodium more in these patients (Additional file 1: Table 3).

There were no significant changes in serum K^+^ concentrations from baseline for all groups throughout the treatment period (Table 5).

### 24-h urine volume and water intake

**Fig. 2:**
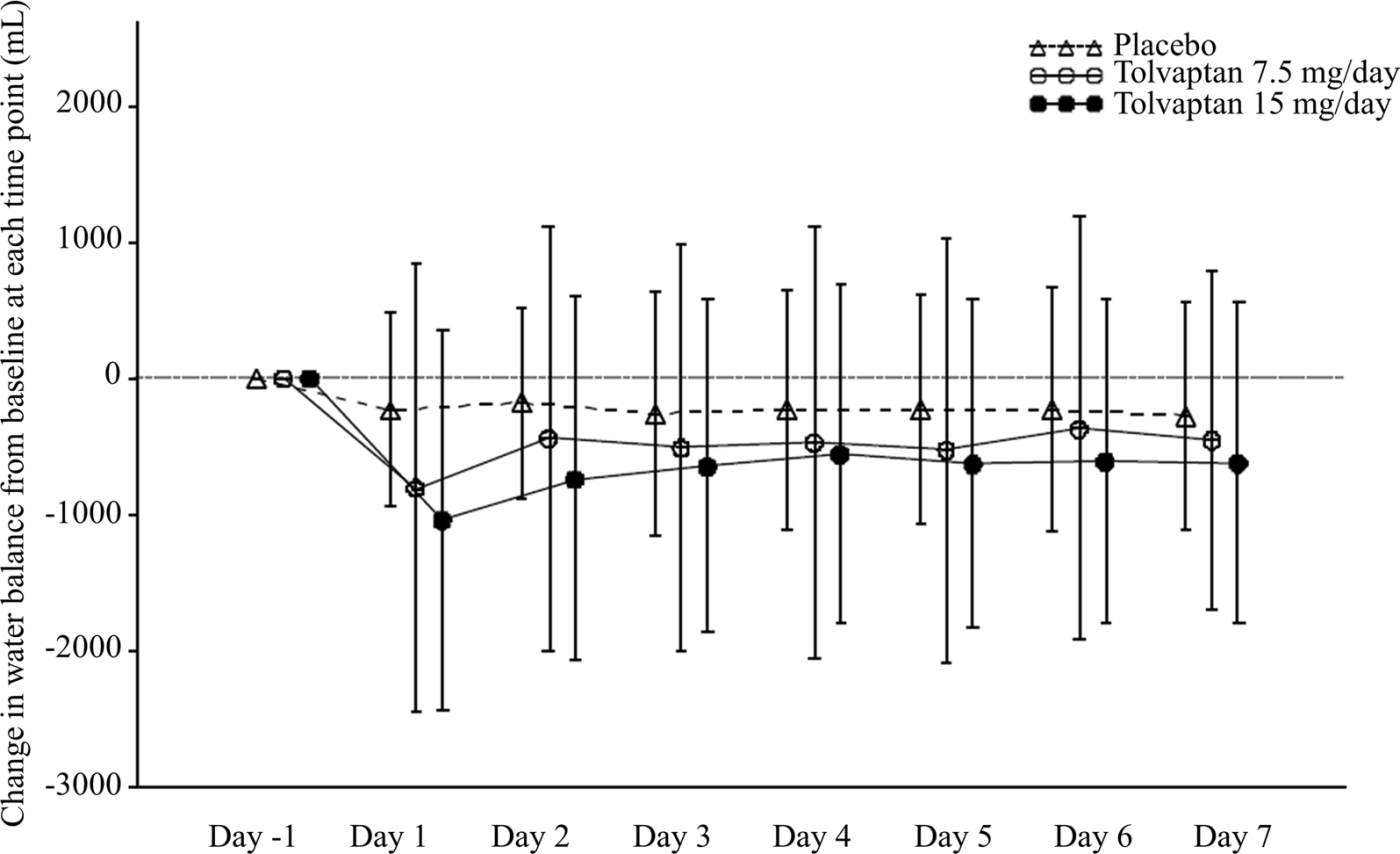
Change in the water balance from baseline at each time point in the placebo, 7.5 mg and 15 mg tolvaptan groups. Data are expressed as the mean ± SD

The 24-h urine volumes in the tolvaptan groups increased from baseline, most obviously at day 1. It is also worth noting that urine output during tolvaptan treatment was dose-dependent (*P* = 0.009 and *P* = 0.004 on day 4 and day 7, respectively). The tolvaptan patient groups also had a higher fluid intake but still a more negative water balance than the placebo group (*P* < 0.001 for both). There was no difference between the tolvaptan groups regarding water balance (Table 6, Fig. 2).

**Table 6:** The 24-h urine volume, fluid intake and water balance of the tolvaptan and placebo groups from baseline to day 7

		Baseline	Day 1	Day 2	Day 3	Day 4	Day 5	Day 6	Day 7	*P *value (versus placebo)
24-h urine volume (mL)	Placebo	1666.9 ± 713.5	2019.5 ± 921.4	1920.9 ± 802.5	2099.0 ± 868.2	1968.7 ± 796.5	2066.0 ± 810.0	1993.5 ± 789.3	1826.6 ± 720.4	–
	Tolvaptan 7.5 mg	1951.8 ± 848.2	3232.5 ± 1565.2	2999.5 ± 1524.0	2890.1 ± 1386.1	2785.6 ± 1430.2	2841.2 ± 1322.5	2748.2 ± 1256.8	2606.5 ± 1245.3	< 0.001
	Tolvaptan 15 mg	1831.4 ± 911.8	3661.0 ± 1791.4	3399.5 ± 1637.1	3139.4 ± 1527.9	2981.8 ± 1402.5	3000.4 ± 1390.7	3019.4 ± 1372.5	2834.9 ± 1312.7	< 0.001
24-h fluid intake (mL)	Placebo	1854.9 ± 708.7	1973.1 ± 782.2	1929.6 ± 767.1	1995.3 ± 839.5	1860.9 ± 806.8	1953.7 ± 791.7	1874.3 ± 802.2	1665.4 ± 727.3	–
	Tolvaptan 7.5 mg	2042.0 ± 1059.5	2505.3 ± 1152.8	2643.7 ± 1255.9	2447.3 ± 1167.9	2381.9 ± 1128.3	2339.4 ± 1087.3	2416.5 ± 1337.0	2175.3 ± 1111.1	< 0.001
	Tolvaptan 15 mg	1953.4 ± 964.6	2745.9 ± 1318.1	2795.4 ± 1369.0	2605.1 ± 1291.5	2537.6 ± 1306.4	2456.6 ± 1237.5	2493.4 ± 1320.4	2295.2 ± 1141.4	< 0.001
Water balance	Placebo	189.8 ± 714.1	− 47.9 ± 746.7	11.9 ± 609.0	− 105.1 ± 793.7	− 105.0 ± 756.1	− 115.0 ± 694.1	− 116.7 ± 768.9	− 155.0 ± 665.1	–
	Tolvaptan 7.5 mg	83.1 ± 1158.7	− 727.1 ± 1352.0	− 364.9 ± 1255.9	− 451.3 ± 1227.9	− 409.5 ± 1174.4	− 498.6 ± 1180.2	− 334.5 ± 1264.2	− 428.8 ± 941.1	< 0.001
	Tolvaptan 15 mg	123.5 ± 873.3	− 921.3 ± 1352.3	− 610.8 ± 1137.8	− 539.0 ± 1069.1	− 447.0 ± 1046.9	− 546.7 ± 1036.3	− 529.7 ± 1039.7	− 553.1 ± 1007.0	< 0.001

### Safety assessments

#### Renal and liver function

Tolvaptan caused significant increases in serum Cr compared with placebo but these increases were far from worsening the renal failure criteria, which is defined as a ≥ 0.3 mg/dL increase in serum Cr. There was no significant difference between the 7.5 mg/day tolvaptan and placebo groups on day 7 (Table 7).

**Table 7:** Comparison of changes in serum creatinine, BUN, TB, AST and ALT on day 4 and day 7 among the 3 groups (SS)

		Placebo (mg/day) (N = 76)		Tolvaptan				*P *value		
				7.5 mg/day (N = 153)		15 mg/day (N = 304)				
		Mean ± SD	Changes from baseline	Mean ± SD	Changes from baseline	Mean ± SD	Changes from baseline	7.5 mg/day tolvaptan versus placebo	15 mg/day mg tolvaptan versus placebo	7.5 mg/day versus 15.0 mg/day tolvaptan
Serum creatinine (mg/dL)	Baseline	0.8 ± 0.2	–	0.8 ± 0.3	–	0.9 ± 0.3	–	–	–	–
	Day 4	0.8 ± 0.2	0.0 ± 0.1	0.9 ± 0.3	0.1 ± 0.2	0.9 ± 0.3	0.1 ± 0.2	0.030	0.001	0.173
	Day 7	0.8 ± 0.2	0.0 ± 0.2	0.9 ± 0.3	0.1 ± 0.2	0.9 ± 0.3	0.1 ± 0.2	0.119	0.019	0.517
BUN (mmol/L)	Baseline	6.2 ± 3.0	–	6.3 ± 3.4	–	6.8 ± 3.6	–	–	–	–
	Day 4	5.8 ± 2.7	− 0.1 ± 1.0	6.2 ± 3.4	0.1 ± 1.6	6.5 ± 3.6	− 0.1 ± 1.7	0.428	0.871	0.434
	Day 7	6.7 ± 3.4	0.5 ± 2.5	6.8 ± 4.0	0.5 ± 2.5	6.7 ± 3.9	0.0 ± 2.0	0.987	0.086	0.027
TB (µmol/L)	Baseline	43.8 ± 42.1	–	49.2 ± 57.2	–	43.6 ± 47.7	–	–	–	–
	Day 4	40.7 ± 37.4	0.6 ± 11.1	45.6 ± 45.0	1.1 ± 13.6	46.4 ± 57.5	2.8 ± 21.0	0.809	0.252	0.317
	Day 7	44.8 ± 52.8	2.6 ± 21.7	48.2 ± 50.4	3.4 ± 23.7	44.6 ± 58.0	0.8 ± 20.2	0.822	0.526	0.260
AST (IU/L)	Baseline	67.4 ± 54.9	–	68.8 ± 56.9	–	66.0 ± 54.2	–	–	–	–
	Day 4	68.6 ± 61.0	3.0 ± 15.2	65.7 ± 55.3	0.2 ± 20.8	65.0 ± 57.9	− 0.6 ± 22.7	0.280	0.133	0.741
	Day 7	68.1 ± 66.2	2.0 ± 18.6	71.0 ± 64.5	4.0 ± 32.7	69.5 ± 66.1	2.2 ± 36.7	0.573	0.951	0.614
ALT/GPT (IU/L)	Baseline	42.2 ± 27.1	–	43.1 ± 37.7	–	44.2 ± 39.3	–	–	–	–
	Day 4	40.5 ± 26.8	− 1.1 ± 8.8	42.0 ± 36.9	0.6 ± 12.8	44.0 ± 35.0	− 0.5 ± 18.5	0.274	0.712	0.467
	Day 7	39.3 ± 26.9	− 1.8 ± 11.6	44.3 ± 40.0	1.9 ± 19.8	45.5 ± 39.5	0.4 ± 30.3	0.087	0.335	0.529

*ALT* alanine transaminase, *AST* aspartate transaminase, *BUN* blood urea nitrogen, *GPT* glutamic-pyruvic transaminase, *Scr* serum creatinine, *TB* total bilirubin

There was no significant increase in the liver function enzymes aspartate transaminase (AST) and alanine transaminase (ALT) or total bilirubin (TB) and BUN for either tolvaptan group compared with the placebo group.

The number of patients who met the definition of Hy’s Law (ALT or AST > 3 × upper limit of normal (ULN) and TB > 2 × ULN) was 1 (1.3%) in the placebo group, 2 (1.3%) in the tolvaptan 7.5 mg/day group and 1 (0.3%) in the tolvaptan 15 mg/day group, with no imbalance among the groups.

#### Adverse events

No significant difference was found in the overall rate of adverse events in the 3 trial groups (Table 8). Adverse events with an incidence of 5% or more were dry mouth and hypokalaemia in all 3 groups, abdominal bloating in only the placebo group (7.9%) and thirst in only the 15 mg tolvaptan group, but not in the placebo or 7.5 mg tolvaptan groups.

**Table 8:** Incidence of adverse events

	Placebo N = 76 (%)	Tolvaptan		*P *value
		7.5 mg/day N = 153 (%)	15 mg/day N = 304 (%)	
AEs observed during the trial	46 (60.5)	98 (64.1)	218 (71.7)	0.084
AEs observed during the treatment	35 (46.1)	81 (52.9)	182 (59.9)	0.065
AEs observed at a rate of ≥ 5% of patients in any group				
Dry mouth	8 (10.5)	25 (16.3)	42 (13.8)	0.483
Abdominal bloating	6 (7.9)	4 (2.6)	11 (3.6)	0.140
Thirst	2 (2.6)	6 (3.9)	35 (11.5)	0.003
Hypokalemia	6 (7.9)	13 (8.5)	21 (6.9)	0.823
Hepatic encephalopathy				0.580
Baseline	1 (1.32)	1 (0.65)	1 (0.33)	
Post-treatment	2 (2.63)	4 (2.61)	2 (0.66)	
Increased number (post-baseline)	1 (1.32)	3 (1.96)	1 (0.33)	
ADRs observed during the trial	9 (11.8)	38 (24.8)	94 (30.9)	0.003
ADRs observed at a rate of ≥ 5% of patients in any group				
Dry mouth	6 (7.9)	25 (16.3)	38 (12.5)	0.188
Thirst	2 (2.6)	6 (3.9)	34 (11.2)	0.005
SADRs	1 (1.3)	1 (0.7)	6 (2.0)	0.543
SAEs observed during the trial	9 (11.8)	10 (6.5)	20 (6.6)	0.262
Upper gastrointestinal bleeding	5 (6.6)	4 (2.6)	4 (1.3)	0.029
Deaths	3 (3.9)	4 (2.6)	8 (2.6)	0.812

All AEs were coded using the Medical Dictionary for Regulatory Activities (MedDRA) 14.0 (Chinese version)

*AEs* adverse events, *ADRs* adverse drug reactions, *SADRs* serious adverse drug reactions, *SAEs* serious adverse events

The rate of adverse drug reactions was higher in the tolvaptan groups; these reactions consisted mainly of thirst and dry mouth. Hypernatraemia occurred in 0%, 0.7% and 1.0% (*P* = 0.667) of patients in the placebo, 7.5 mg and 15 mg tolvaptan groups, respectively. Interestingly, upper gastrointestinal bleeding occurred to a lesser extent in the high tolvaptan dosage group (*P* = 0.029). However, since this study excluded any patients who had potential risks of bleeding during treatment and the total number of cases was low, any conclusions should be cautiously drawn.

No notable abnormalities were detected regarding vital signs and 12-lead electrocardiograms, and there were no serious adverse drug reactions in any of the patient groups.

In this study, a total of 15 patients died: 3/76 (3.9%), 4/153 (2.6%), and 8/304 (2.6%) died in the placebo, 7.5 mg tolvaptan and 15 mg tolvaptan groups, respectively. All deaths occurred after the treatment period and were determined to be unrelated to the trial drug (Additional file 1: Table 4).

## Discussion

This clinical trial was a multicentre, randomized, placebo-controlled, double-blind study comprising the largest possible sample size for patients being treated with tolvaptan for hepatic cirrhosis. This confirmed the results of earlier studies showing that ascites in cirrhosis patients who did not respond to conventional diuretic therapy could be improved by tolvaptan therapy [11, 12, 14]. The improvement in ascites was mainly reflected in changes in body weight and abdominal circumference. In Japan, a phase III study used computer tomography (CT) to calculate ascites volume as the secondary endpoint, but this technology has not been generally adopted in China. Abdominal circumference has been widely used in China for decades, especially in grassroots hospitals, has a good correlation with body weight changes and ascites volume [13], and actually has more value for guidance in real-world clinical practice in China [15].

In the present study, doses of both 15 mg and 7.5 mg per day were effective, and significant differences were not observed among the dosage groups or in renal function indicators. The serum Cr concentration increased in both tolvaptan dose groups in the present trial, but it was more obvious in the 15 mg tolvaptan treatment group. Therefore, a relatively low dose (7.5 mg/day) of tolvaptan may be an optimal and safe treatment for cirrhotic patients with ascites. However, the Japanese ascites guidelines recommend starting with 3.75 mg/d and showed that tolvaptan at 3.75 mg/day exerts some effects, but 7.5 mg/day may be more beneficial [16]. Based on a continuous but consistent decrease in body weight and great improvement in the ascites volume at 7.5 mg/day, it is unlikely that 3.75 mg/day should be the clinical choice for Chinese ascites patients, but further clinical trials are needed to unequivocally confirm this view.

Cirrhosis, especially in advanced stages, is associated with a decrease in plasma albumin, and low albumin levels play a role in the formation of ascites. One of the functions of albumin is to enhance the diuretic effect of furosemide. However, studies have shown that combination therapy with loop diuretics and albumin increased urine output for the first 8 h, an effect that was no longer significant after 24 h [17]. This study is consistent with others since tolvaptan was shown to have good efficacy in patients with liver cirrhosis, with regard to both low and high serum albumin concentrations [18]. The present study showed that patients with lower albumin levels had even greater body weight reductions. This negative correlation trend was found in a pilot study. A possible explanation is that patients with lower albumin concentrations had more serous volume overload [19].

In our study, the baseline sodium concentration of 143 (27%) patients was below 135 mmol/L. For both the overall population and hyponatraemia subgroup, placebo further decreased the sodium level, while a low sodium level was an independent risk factor for poor prognosis in cirrhosis patients, and restoration was related to significantly improved 6-month survival rates [20]. Thus, tolvaptan is beneficial to both hyponatraemia and normal natraemia patients. It is also noteworthy that although both tolvaptan groups had increased sodium serum concentrations, only the 15 mg dosage group reached overall sodium normalization for hyponatraemia patients. It is possible that hyponatraemia patients need dose adjustments during treatment, as reported in the SALT study [21].

The occurrence of hepatic injury in 3 patients with autosomal dominant polycystic kidney disease who were treated with tolvaptan in a double-blind placebo-controlled trial [22, 23] led the FDA to worry about its safety in patients with liver disease [24]. However, an independent, hepatic adjudication committee reviewed the data from autosomal dominant polycystic kidney disease (ADPKD) and non-ADPKD tolvaptan trials and concluded that no imbalance in hepatic events was observed between the tolvaptan and placebo groups in lower-dose clinical trials of patients with cirrhosis, hyponatraemia or heart failure [25]. The present study also did not find any imbalance in liver function between the tolvaptan groups and the placebo group.

Responses to tolvaptan treatment not only reflect its short-term effectiveness but are also linked to significantly improved overall survival of patients with cirrhotic livers. This action was independent of the response definition or the presence of hepatocellular carcinoma [26, 27]. Different studies have identified different factors to predict tolvaptan responsiveness, such as urinary excretion of aquaporin 2, free water clearance, urinary sodium excretion, portal vein pressure, the BUN/Cr ratio, urine Na^+^/K^+^ ratio, etc. [28–32]; the most commonly identified factor was the baseline BUN level. The definitions of response were not the same in these studies, from increasing 500 mL urine to reducing 2 kg body weight. Some of the studies further explored the cut-off value of baseline BUN levels, and although the cut-off values were not the same, they were very close (from 22.4 to 29.0 mg/dL) [33–39]. This study set body weight loss equal to or greater than 1.5 kg in 7 days as the response criteria and verified that the baseline BUN level was a predictive factor.

The present trial had several limitations. First, the determination of clinically meaningful parameters (e.g., ascites volume and ascites-related symptoms) may be required to evaluate any future treatment of cirrhotic patients with tolvaptan. Second, several evaluations were conducted during only short-term treatment with tolvaptan.

## Conclusions

Tolvaptan at doses of 7.5 mg/day or 15 mg/day significantly reduced the body weight and abdominal circumference of cirrhotic patients with ascites. Our findings indicate that 7.5 mg/day tolvaptan may be an optimal initial dose for Chinese cirrhotic patients with ascites who responded poorly to conventional diuretic therapy.

## Supplementary information

**Additional file 1**. Exclusion criteria of the study.

## Data Availability

The datasets used and/or analyzed during the current study are available from the corresponding author on reasonable request.

## Abbreviations

ADPKD: Autosomal dominant polycystic kidney disease
ALT: Alanine transaminase
AST: Aspartate transaminase
BUN: Blood urea nitrogen
Cr: Creatinine
CT: Computer tomography
RCT: Randomized controlled trial
TB: Total bilirubin
ULN: Upper limit of normal
