# Supplementary material for: Tolvaptan therapy of Chinese cirrhotic patients with ascites after insufficient diuretic routine medication responses: a phase III clinical trial
Source: BMC Gastroenterol. 2020 Nov 19;20:391. doi: 10.1186/s12876-020-01536-0 (PMC7678173; doi:10.1186/s12876-020-01536-0)
Supplement: Supplementary file 1 — Additional file 1. Exclusion criteria of the study. [file 12876_2020_1536_MOESM1_ESM.docx]

**Supplementary Table 1. Changes in body weight (kg) from baseline at each time point in the placebo (0 mg), 7.5 mg and 15 mg tolvaptan groups**

|  | **Placebo (mg) (N = 76)** | |  | **Tolvaptan** | | | | |  | ***P-*value** | | |
| --- | --- | --- | --- | --- | --- | --- | --- | --- | --- | --- | --- | --- |
|  |  |  |  | **7.5 mg/day (N = 153)** | | | **15 mg/day (N = 301)** | |  |  |  |  |
|  | Mean ± SD | Changes from baseline |  | Mean ± SD | Changes from baseline | | Mean ± SD | Changes from baseline |  | 7.5 mg Tolvaptan *vs* placebo | 15.0 mg Tolvaptan *vs* placebo | 7.5 mg *vs* 15.0 mg Tolvaptan |
| Body weight changes | | | | | | | | | | | | |
| Baseline | 63.5 ± 12.8 |  | | 60.6 ± 10.1 | - | | 62.9 ± 12.0 | - | | < 0.001 | < 0.001 | 0.016 |
| Day 1 | 63.3 ± 12.7 | -0.2 ± 1.2 | | 59.6 ± 10.1 | -0.9 ± 1.2 | | 61.7 ± 11.9 | -1.2 ± 1.1 | | < 0.001 | < 0.001 | 0.092 |
| Day 2 | 63.1 ± 12.8 | -0.4 ± 1.3 | | 59.3 ± 10.2 | -1.2 ± 1.5 | | 61.5 ± 11.9 | -1.5 ± 1.5 | | < 0.001 | < 0.001 | 0.709 |
| Day 3 | 62.9 ± 12.7 | -0.6 ± 1.5 | | 59.0 ± 10.1 | -1.5 ± 1.9 | | 61.3 ± 11.9 | -1.6 ± 1.8 | | 0.014 | < 0.001 | 0.194 |
| Day 4 | 62.6 ± 12.7 | 0.9 ± 1.6 | | 59.0 ± 10.2 | -1.6 ± 2.0 | | 61.1 ± 11.8 | -1.8 ± 2.0 | | 0.052 | 0.003 | 0.301 |
| Day 5 | 62.4 ± 12.7 | -1.1 ± 1.9 | | 58.9 ± 10.1 | -1.7 ± 2.1 | | 60.9 ± 11.9 | -1.9 ± 2.1 | | 0.034 | 0.002 | 0.325 |
| Day 6 | 62.4 ± 12.4 | -1.1 ± 2.1 | | 58.8 ± 10.3 | -1.8 ± 2.4 | | 60.9 ± 11.9 | -2.0 ± 2.2 | | 0.026 | 0.001 | 0.339 |
| Day 7 | 62.3 ± 12.4 | -1.2 ± 2.2 | | 58.6 ± 10.1 | -2.0 ± 2.4 | 60.8 ± 12.0 | | -2.2 ± 2.5 | | 0.625 | 0.662 | 0.202 |
| EOT^*^ | 61.6 ± 12.7 | -2.4 ± 3.5 | | 58.3 ± 9.7 | -2.1 ± 4.2 | 60.2 ± 11.6 | | -2.6 ± 2.9 | | < 0.001 | < 0.001 | 0.016 |

EOT: end of treatment

**Supplementary Table 2. Factors associated with the treatment effect of tolvaptan (body weight loss > 1.5 kg) analysed by a generalized linear regression model**

| **Variable** | | **OR** | **95% CI** | ***P*-value** |
| --- | --- | --- | --- | --- |
| Treatment group | Placebo | 1.000 |  |  |
|  | Tolvaptan 7.5 mg/day | 1.823 | 1.0 - 3.2 | 0.036 |
|  | Tolvaptan 15 mg/day | 2.132 | 1.3 - 3.6 | 0.004 |
| Gender | Male | 1.000 |  |  |
|  | Female | 0.909 | 0.6 - 1.3 | 0.618 |
| Age (years) |  | 1.002 | 1.0 - 1.0 | 0.780 |
| Body weight (baseline, kg) |  | 1.018 | 1.0 - 1.0 | 0.019 |
| Abdominal circumference (cm) |  | 1.015 | 1.0 - 1.0 | 0.066 |
| Duration of cirrhosis |  | 1.000 | 1.0 - 1.0 | 0.400 |
| Etiology of liver cirrhosis | | | | |
| Hepatitis B | No | 1.000 |  |  |
|  | Yes | 0.774 | 0.5 - 1.1 | 0.165 |
| Hepatitis C | No | 1.000 |  |  |
|  | Yes | 1.115 | 0.6 - 2.2 | 0.753 |
| Alcoholic hepatitis | No | 1.000 |  |  |
|  | Yes | 1.419 | 0.9 - 2.2 | 0.122 |
| Primary biliary cirrhosis | No | 1.000 |  |  |
|  | Yes | 1.035 | 0.3 - 3.1 | 0.952 |
| Liver cancer | No | 1.000 |  |  |
|  | Yes | 0.664 | 0.4 - 1.1 | 0.117 |
| Child-Pugh grade | Grade C | 1.000 |  |  |
|  | Grade B | 0.958 | 0.7 - 1.4 | 0.924 |
|  | Grade A | 0.860 | 0.2 - 3.1 | 0.839 |
| Hepatic encephalopathy | No | 1.000 |  |  |
|  | Yes | > 999.999 | < 0.001 - > 999.999 | 0.984 |
| Ascites | Mild | 1.000 |  |  |
|  | Moderate | 1.255 | 0.8 - 2.0 | 0.333 |
| Albumin level (g/dL) | > 3.5 | 1.000 |  |  |
|  | 2.8 - 3.5 | 1.425 | 0.9 - 2.3 | 0.947 |
|  | < 2.8 | 1.983 | 1.1 - 3.4 | 0.016 |
| Scr (baseline, mg/dL) |  | 0.996 | 1.0 - 1.0 | 0.202 |
| UA (baseline, μmol/L) |  | 0.999 | 1.0 - 1.0 | 0.056 |
| BUN (baseline, mmol/L) |  | 0.942 | 0.9 - 1.0 | 0.020 |
| eGFR (baseline, mL/min/1.73 m^2^) |  | 1.000 | 1.0 - 1.0 | 0.289 |

Note: BUN, blood urea nitrogen; eGFR, estimated glomerular filtration rate; Scr, serum creatinine; UA, uric acid.

**Supplementary Table 3.** **The changes in serum sodium concentrations from baseline to day 7 in patients with and without hyponatraemia**

|  | **Patients with hyponatremia** | | | **Patients without hyponatremia** | | |
| --- | --- | --- | --- | --- | --- | --- |
|  | **Placebo (mg/day)**  **N = 17** | **Tolvaptan**  **7.5 mg/day**  **N = 41** | **Tolvaptan 15 mg/day**  **N = 84** | **Placebo (mg/day)**  **N = 58** | **Tolvaptan**  **7.5 mg/day**  **N = 109** | **Tolvaptan**  **15 mg/day**  **N = 215** |
| Baseline | 131.3 ± 3.1 | 130.2 ± 4.5 | 130.9 ± 3.7 | 139.4 ± 2.7 | 139.2 ± 2.7 | 139.2 ± 2.8 |
| Day 7 | 130.0 ± 4.1 | 133.2 ± 5.0 | 135.0 ± 4.9 | 138.9 ± 3.2 | 140.1 ± 3.5 | 140.6 ± 3.4 |
| Day 7 - baseline | -2.0 ± 3.7 | 2.5 ± 3.0^△^ | 3.9 ± 5.0^△^ | -0.5 ± 3.2 | 0.9 ± 3.3^△^ | 1.4 ± 3.3^△^ |
| Difference from placebo, 95% CI | - | -4.5 (-6.0 - -2.4) | -5.9 (-8.8 - -3.0) | - | -1.4 (-2.5- -0.3) | -1.9 (-2.9 - -0.9) |

Note: Hyponatremia patients: Na^+^ < 135 mmol/L

△: Compared to placebo significant difference (*P* < 0.05)

**Supplementary Table 4. Information on subjects who died (SS)**

| **Group** | **Adverse events** | **Relevance to the investigational drug** | **Outcome** |
| --- | --- | --- | --- |
| Tolvaptan 7.5 mg/day | Critical illness caused by compensated and decompensated alcoholic liver cirrhosis | Possibly unrelated | Death |
| Tolvaptan 7.5 mg/day | Upper gastrointestinal hemorrhage | Unrelated | Death |
| Tolvaptan 7.5 mg/day | Kidney failure | Unrelated | Death |
| Tolvaptan 7.5 mg/day | Progressive heart failure | Unrelated | Death |
| Tolvaptan 15 mg/day | Lymphatic metastasis in gastric cancer | Unrelated | Death |
| Tolvaptan 15 mg/day | Death (epatorenal syndrome) | Unrelated | Death |
| Tolvaptan 15 mg/day | Intraperitoneal hemorrhage, hemorrhagic shock | Possibly unrelated | Death |
| Tolvaptan 15 mg/day | Advanced primary liver cancer | Unrelated | Death |
| Tolvaptan 15 mg/day | Upper gastrointestinal hemorrhage | Unrelated | Death |
| Tolvaptan 15 mg/day | Progression of primary liver cancer | Unrelated | Death |
| Tolvaptan 15 mg/day | Aggravation of renal and liver function | Possibly unrelated | Death |
| Tolvaptan 15 mg/day | Multiple system organ failure | Possibly unrelated | Death |
| Placebo | Hepatic failure | Unrelated | Death |
| Placebo | Progression of primary liver cancer | Unrelated | Death |
| Placebo | Aggravation of lower white blood cell count (agranulocytosis) and electrolyte disturbance | Possibly unrelated | Death |

**Supplementary File 1.** Exclusion criteria of the study

Patients who had any of the following indications were ineligible for enrollment: hepatic encephalopathy (hepatic coma ≥ grade II including symptoms of somnolence to semi stupor but responsive to verbal stimuli, confusion, gross disorientation; and in grade IV, coma with patient failure to respond to noxious or verbal stimuli, malignant ascites, uncontrolled spontaneous bacterial peritonitis, alimentary tract hemorrhage within 10 days prior to screening, serum creatinine > 1.5 times normal, serum Na^+^>145mEq/L, serum K^+^ > 5.5mEq/L, Child-Pugh score > 12, and those treated with blood products containing albumin within 4 days prior to the initiation of the trial treatments.


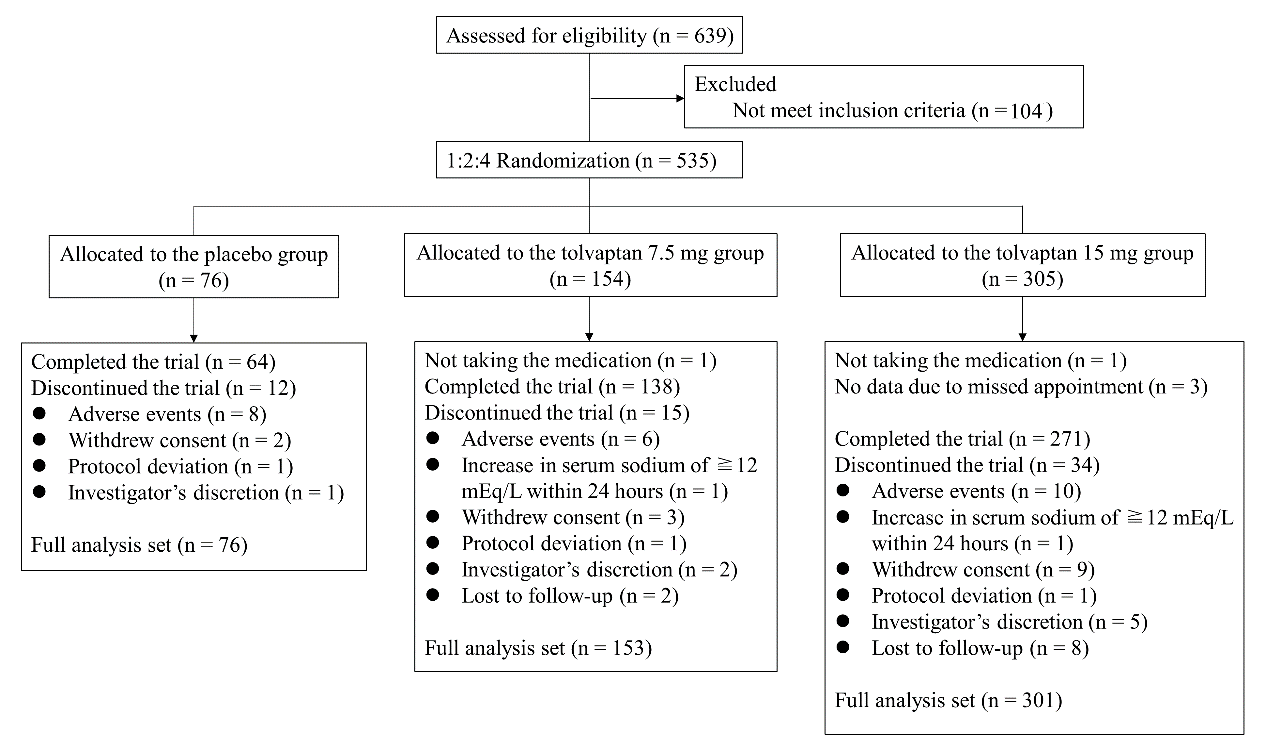


**Supplementary Figure 1. Schematic diagram of patient screening**
